# The a.c. Josephson effect without superconductivity

**DOI:** 10.1038/ncomms7524

**Published:** 2015-03-13

**Authors:** Benoit Gaury, Joseph Weston, Xavier Waintal

**Affiliations:** 1 Université Grenoble Alpes, INAC-SPSMS, F-38000 Grenoble, France; 2 CEA, INAC-SPSMS, F-38000 Grenoble, France

## Abstract

Superconductivity derives its most salient features from the coherence of the associated macroscopic wave function. The related physical phenomena have now moved from exotic subjects to fundamental building blocks for quantum circuits such as qubits or single photonic modes. Here we predict that the a.c. Josephson effect—which transforms a d.c. voltage *V*_b_ into an oscillating signal cos (2*eV*_b_*t*/*ħ*)—has a mesoscopic counterpart in normal conductors. We show that when a d.c. voltage *V*_b_ is applied to an electronic interferometer, there exists a universal transient regime where the current oscillates at frequency *eV*_b_/*h*. This effect is not limited by a superconducting gap and could, in principle, be used to produce tunable a.c. signals in the elusive 0.1–10-THz ‘terahertz gap’.

## Abstract (web summary)

Most of the peculiar effects resulting from superconductivity have counterparts in nonsuperconducting nanoelectronic devices, but not yet in the a.c. Josephson effect. Here, the authors propose how to generate a transient version of this phenomenon in a normal conductor by abruptly changing the bias voltage.

## Graphical abstract

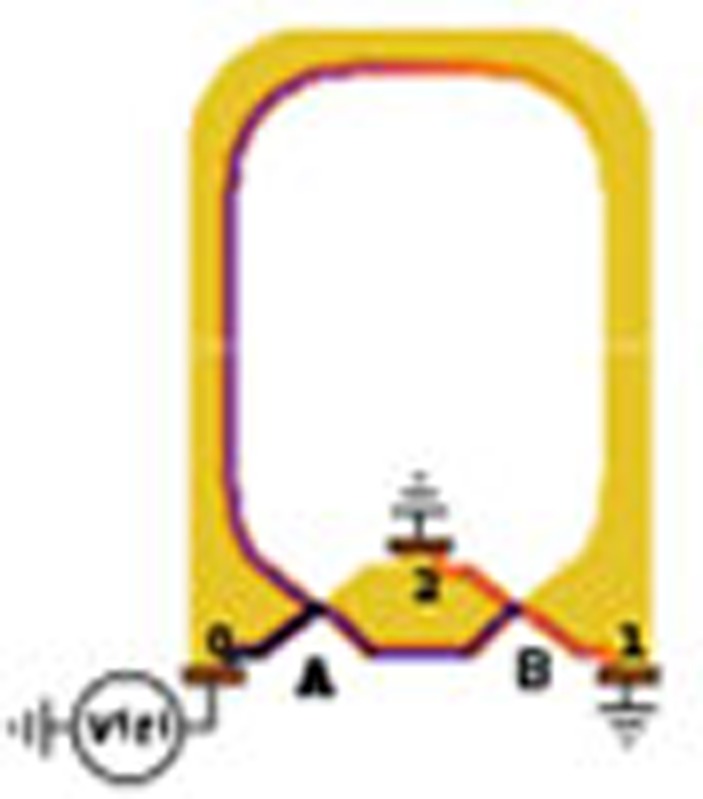

Superconductivity, a macroscopic quantum state, is described by a wavefunction whose phase is physically significant. Indeed, quantum mechanical interference effects are ubiquitous in superconducting systems, similar to those observed at the microscopic scale in atomic physics. Since the 80s, such effects have also been observed at the mesoscopic (or nano) scale in condensed matter. Most of the peculiar effects observed in superconductors have an analogue in ‘normal’ quantum nanoelectronics: the d.c. SQUID (superconducting quantum interference device) corresponds to the Aharonov–Bohm effect^1^, supercurrents (at the origin of the Meissner effect) correspond to the so-called persistent currents^2^,^3^,^4^. The a.c. Josephson effect in superconductors is perhaps the most striking manifestation of these interference effects at a macroscopic scale^5^; a d.c. voltage bias *V*_b_ applied across a weak link between two superconductors creates an oscillating current with frequency 2*eV*_b_/*h*. This voltage to frequency conversion is used in metrology to define the volt in terms of the second^6^, as well as in a wealth of superconducting devices (radiofrequency (RF)-SQUIDs, quantum bits)^7^. Its origin is rather straightforward. The energy of the left superconductor *eV*_b_ is higher than the right one, so that its wavefunction gets an extra oscillating factor 
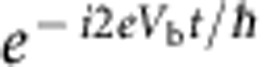
. The junction produces an interference between these two wavefunctions, hence the oscillations.

The absence of a corresponding mesoscopic effect in normal conductors is in itself surprising from a theoretical perspective; superconductivity is well described by the Bogoliubov–De Gennes equation^8^, a simple extension of the Schrödinger equation. Here we report on this missing effect. We find that an oscillating signal cos (*eV*_b_*t*/*ħ*) is generated in the transient regime that follows an abrupt change in the bias voltage applied to a normal conductor.

## Results

### Mach–Zehnder interferometer

**Figure 1:**
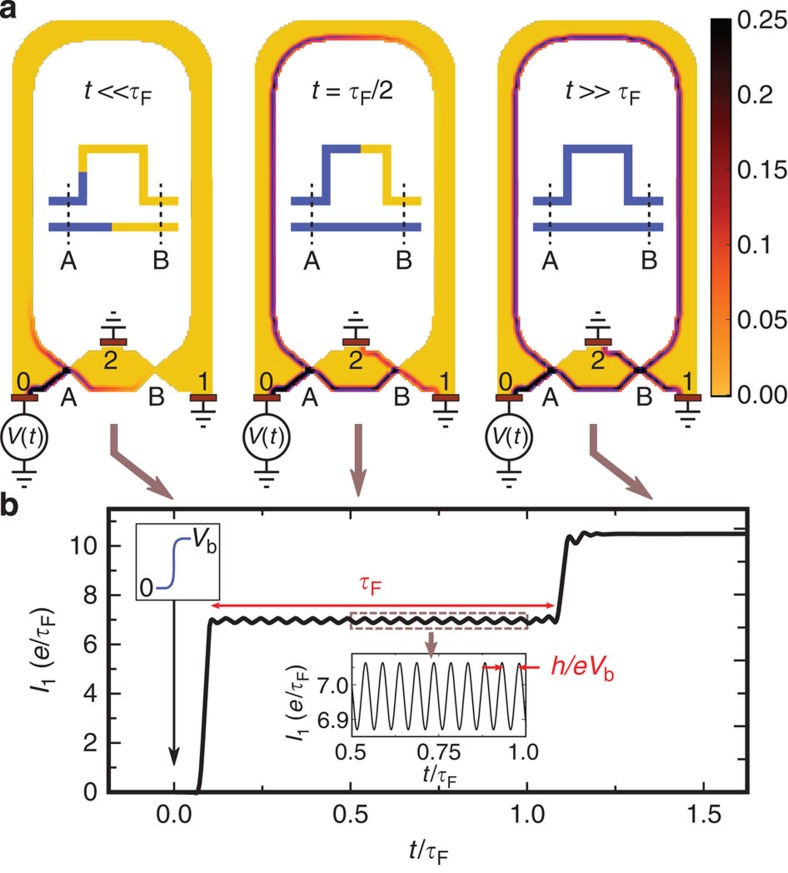
Raising the d.c. voltage bias on a Mach–Zehnder interferometer in the quantum Hall regime. (**a**) Colour plot of the local electronic charge density in unit of 10^11^ cm^−2^ (measured from equilibrium) in a three terminal Mach–Zehnder interferometer in the quantum Hall regime. The simulation was performed considering a two-dimensional electron gas of density *n*_*s*_=10^11^ cm^−2^, corresponding to a Fermi energy *E*_*F*_=3.47 meV or equivalently to a Fermi wavelength *λ*_*F*_=79 nm. At *t*=0, the voltage bias is raised from *V*(*t*<0)=0 to *V*_b_=20*h*/(*eτ*_F_). The three colour plots correspond to three snapshots for different times as indicated by the arrows. A two-dimensional electron gas (yellow) is connected to the three electrodes, the semi-transparent quantum point contacts A and B act as beam-splitters. Insets: schematics of the propagation of the voltage bias along the two arms of the interferometer. (**b**) Transmitted current at contact 1. Upper inset: schematic of the raising of the bias voltage. Lower inset: zoom on the oscillations of the current.

Figure 1a sketches a—two path—electronic Mach–Zehnder interferometer. This device, implemented in a two-dimensional gas under high magnetic field, has lately become a rather standard tool of the mesoscopic physicist^9^,^10^. In the quantum Hall regime the bulk of the electronic gas is insulating and the electronic propagation only occurs on the edges of the sample. One can realize electronic beam-splitters with quantum point contacts, and in this way ensure that only two paths are available for any travelling electrons. The sample is very asymmetric, the upper arm being much longer than the lower one, which implies an extra time of flight *τ*_F_=*L*/*v*_g_ (with *L* the extra length of the upper arm with respect to the lower one and *v*_*g*_ the group velocity of the edge state). At *t*=0 one raises the bias voltage applied on contact 0 from *V*(*t* <0)=0 to *V*(*t*>*τ*_P_)=*V*_b_. While the exact manner in which the voltage is raised is unimportant, the rise time *τ*_P_ must be sufficiently fast (*τ*_P_<*τ*_F_) and the voltage drop spatially sharp enough (compared to *L*)^11^,^12^. Figure 1b shows the transmitted current *I*_1_(*t*) as a function of time *t*, and we can discern three distinct regimes. In the beginning (Fig. 1a left) the voltage pulse did not have enough time to propagate up to contact 1, and *I*_1_(*t*)=0. During a transient regime of duration *τ*_F_ (Fig. 1a middle), the pulse has arrived at contact 1 from the lower arm but not yet from the upper one. The current increases to a finite value. Finally (Fig. 1a right), the pulse arrives from the upper arm and the current increases to its stationary value. The most noteworthy feature of Fig. 1b lies in the transient regime; the current oscillates with frequency *eV*_b_/*h* around a d.c. component. This transient oscillatory regime is the mesoscopic analogue of the a.c. Josephson effect. It is to the a.c. Josephson effect what persistent currents^2^ are to supercurrents.

Let us start by discussing this transient oscillatory regime at the qualitative level. At equilibrium there is no net current flowing in the system. However, a crucial point is to recognize that in conductors this is due to the compensation between the current coming from the various electrodes, not to the absence of motion altogether. In other words, before we switch on the voltage bias there already are electronic waves in the system, which consist of the coherent superposition of the lower and upper path. This is in sharp contrast with, say, an optical experiment where one would suddenly switch on a laser beam. Raising the bias voltage should be understood as a modification of the energy of an existing wave (rather than the sudden injection of electrons in vacuum). This ‘modification’ of the energy propagates through the system ballistically. In the transient regime (where the corresponding front has travelled through the lower path but not yet through the upper one), the amplitude of the lower path acquires an extra 
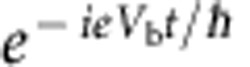
 phase with respect to the upper one. As a result, the current (which is proportional to the probability associated to the total amplitude) oscillates in time.

The theory required to describe the transient oscillatory regime follows naturally from the above picture. Within the time-dependent scattering approach^11^, one finds that the wavefunction close to contact 0 is a plane wave that acquires an additional phase when the bias voltage is raised,


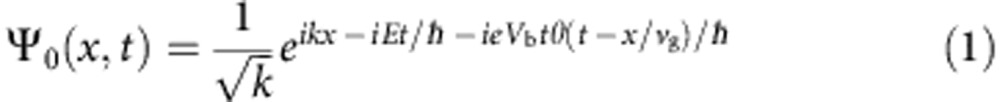


where *θ*(*x*) is the Heaviside function, *E* is the incident energy of the electron, *k* the corresponding momentum and the curved coordinate *x* follows the edge of the sample. We have assumed for simplicity a linear dispersion relation *E*(*k*)=*ħv*_g_*k* and the condition 
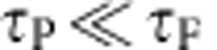
. We see from equation (1) that raising the voltage induces an oscillating phase difference 
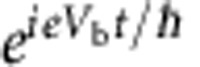
 between the front and the rear of the wave. One can consider this phase difference as the time-dependent extension of the stationary case that was discussed in refs 12, 13. The device uses the delay time *τ*_F_ between the two arms to create an interference between the rear and the front of the wavefunction, generating the oscillatory behaviour. In the transient regime, the wavefunction close to contact 1 is the superposition of the contributions from the two paths and one finds,


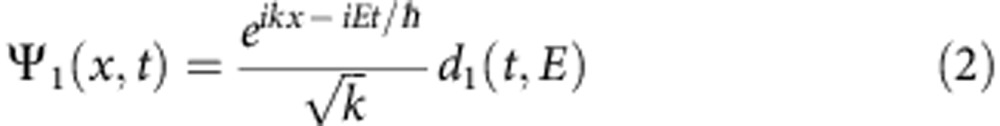


with the total time-dependent transmission amplitude *d*_1_(*t*, *E*) given by,


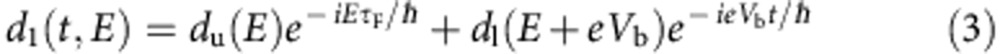


The amplitudes *d*_u/l_ for the upper/lower arm are given in terms of the transmission probabilities *T*_A/B_ of the quantum point contacts, 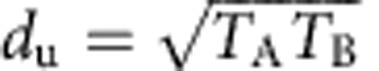 and 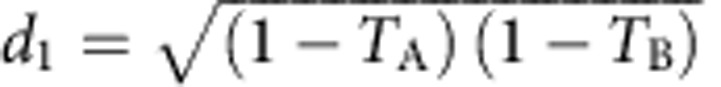. Using the time-dependent generalization of the Landauer formula^11^,


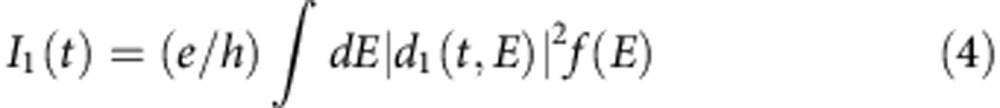


[*f*(*E*) is the Fermi function, equation (4) includes the equilibrium current injected from contact 0 which needs to be subtracted], we finally get the current at contact 1 during the transient regime,


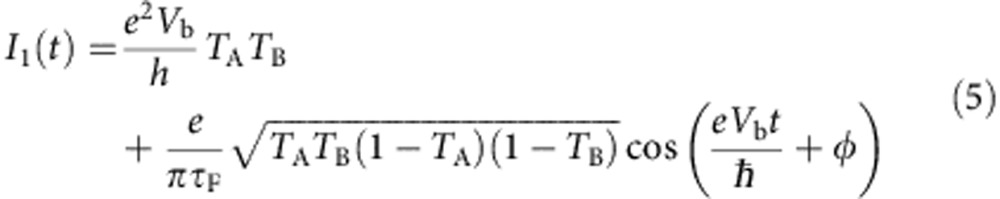


Equation (5) is the main result of this article and agrees with the direct microscopic numerical calculations presented above. While the precise coefficients depend on the particular interferometer considered, its structure is totally general. It contains a d.c. term plus an a.c. term at frequency *eV*_b_/*h*; the amplitude of the a.c. current is of the order of *e*/*τ*_F_. For a typical micrometre sized Mach–Zehnder interferometer, the amplitude of the a.c. current is of the order of a few nA.

### Fabry–Perot cavity

**Figure 2:**
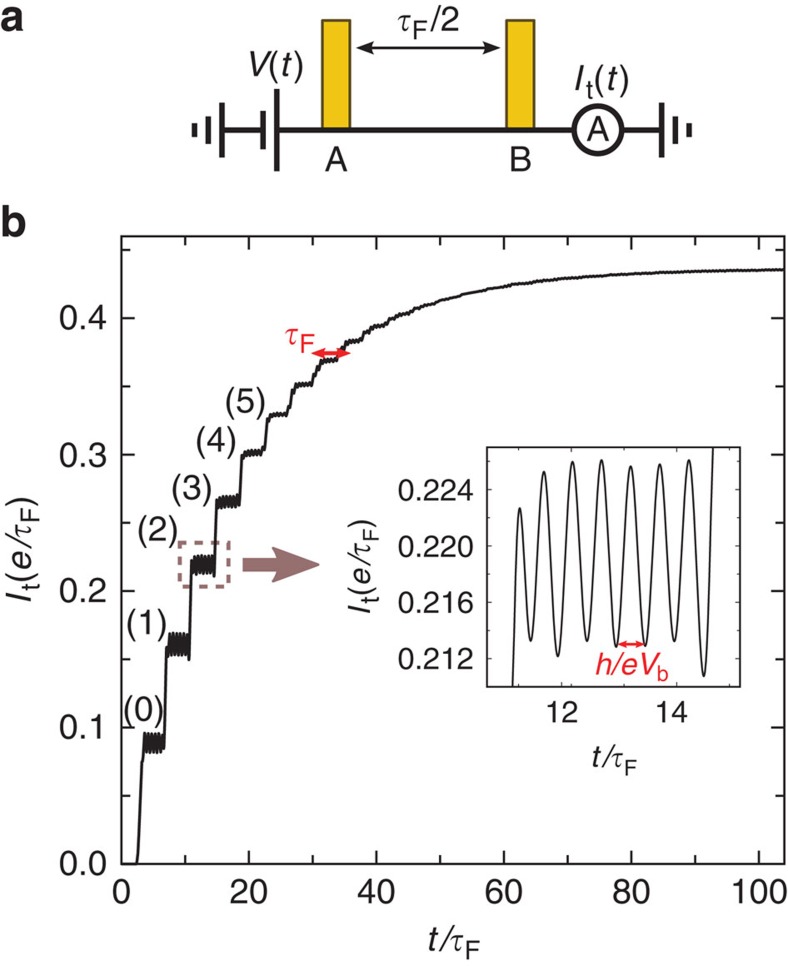
Raising the d.c. voltage bias on a Fabry–Perot interferometer. (**a**) Schematic of the Fabry–Perot cavity (*T*_A_=*T*_B_=0.1). (**b**) Transmitted current (in units of *e*/*τ*_F_, where *τ*_F_ is twice the time of flight between the two barriers) as a function of time for a Fabry–Perot cavity. At *t*=0, the voltage bias is raised from *V*(*t*<0)=0 to *V*_b_=6*h*/(*eτ*_F_). Inset: zoom on the oscillations of the current on a plateau.

The Mach–Zehnder interferometer is simple conceptually, but challenging experimentally in terms of the lithography (for the central electrode 2), low temperature and high magnetic field. Fabry–Perot cavities, in contrast, are ubiquitous and occur every time two barriers are put in series. Examples include carbon nanotubes^14^, quantum Hall systems^15^ and semi-conducting nanowires^16^. Figure 2 shows a sketch of the Fabry–Perot geometry together with a numerical calculation of the measured current as a function of time. The *I*_t_(*t*) curve now features many steps that correspond to the arrival of the path with direct transmission (0), the path with one reflection on B and A (1), two reflections (2) and so on. Again, each of these steps is accompanied by oscillations at the frequency *eV*_b_/*h*. On decreasing the transparencies of the barriers, *T*_A_ and *T*_B_, the Fabry–Perot resonances gradually become true bound states and the duration of the transient regime increases accordingly. This situation is very close, mathematically, to the true Andreev bound states that occur in a Josephson junction^17^.

## Discussion

Although the practical analytical and numerical calculations performed above have been made for specific geometries, one immediately realizes that the existence of transient a.c. oscillations is universal; it must be present in any coherent system where >1 path contributes to transport. Examples include electronic interferometers (Mach–Zehnder, Fabry–Perot, Aharonov–Bohm) but also disordered systems (universal conductance fluctuations) or in fact any electronic resonance in a wide spectrum of devices and materials. Although its magnitude and duration will strongly depend on the particular geometry studied, its frequency—*eV*_b_/*h*—will not, providing a unique signature for its presence. Let us note that the terminology of ‘(mesoscopic) a.c. Josephson effect without superconductivity’ has been used in the past^18^ but with a very different meaning. In ref. 18, the frequency of the oscillation is proportional to the rate at which one varies a magnetic field through a loop. In contrast, here the raising time of the voltage can be arbitrarily slow compared with the oscillating frequency. In particular, in opposition to its superconducting counterpart, the a.c. frequency predicted here is not limited by a superconducting gap, meaning that high frequencies can be obtained. The effect could possibly be used to make tunable radiofrequency sources that reach the THz regime and beyond.

Experimentally, the field of ultrafast quantum transport is developing at an ever-increasing pace. Recent examples include the demonstration of single-electron sources resolved in energy^19^ or time^20^ and measurements in the THz range^21^. At the same time, important progress has been made in optics in the far infrared down to frequencies of a few THz, so that the ‘terahertz gap’ is rapidly shrinking^22^. Being able to manipulate electrons in the THz range will open a wealth of new possibilities: interferences at high (possibly room) temperature, new regimes where photonic and fermionic excitations share the same frequency range and new effects based on the dynamical aspects of quantum mechanics, such as the one proposed here.

As argued above, the present proposal can be implemented in a wide variety of coherent nanoelectronic devices as well as with many different measurement setups (and corresponding frequencies). Let us end this letter with a discussion of the different possibilities to measure it experimentally. The requirements to observe the transient a.c. oscillations are threefold. First, the temperature must be low enough for the phase coherence length to be larger than the system size (more precisely longer than the longest path involved). This condition translates into a simple operational one, one must observe interference patterns in the sample using d.c. measurements—the a.c. requirement is not more stringent than the usual d.c. one. Second, one needs to be able to raise the potential faster than the time of flight *τ*_F_ so that a fast pulse generator is needed. Pulse generators with raise time as fast as 100 ps can be found commercially while 10 ps is available in the lab. On chip complementary metal-oxide-semiconductor-based ring oscillators might also be possible alternative methods to produce such a fast train of square pulses. Finally, one must be able to measure fast enough to access the frequency *eV*_b_/*h*, which itself must be >1/*τ*_F_ in order for several oscillations to take place within the transient regime. In practice, one would periodically switch on and off the bias voltage *V*_b_ to ‘repeat’ the experiment and accumulate the a.c. signal. Ideally, one would measure the time-dependent current in a ‘coherent’ way, that is, one would directly measure the current as a function of time and observe the transient regime. This is, however, probably difficult as it requires an experimental setup with very high bandwidth and very fast acquisition of the signal. The measurement could also be performed on chip by coupling the system to, say, a qubit as the signal produced by the transient a.c. effect is very close to the one used to produce Rabi oscillations. In any case these ‘coherent’ measurements are not easy and also not adapted to very high frequencies (photons in the optical to far infrared frequencies). A second strategy is to measure the signal ‘incoherently’, that is, measure the spectral density of the current *S*_*I*_(*ν*) or equivalently the number of photons *N*(*ν*)=*Z*_c_*S*_I_(*ν*)/*hν* (*Z*_*c*_: electromagnetic impedance of the system) with, for example, quadratic detectors (RFs) or photon detectors (optical frequencies)^23^. The transient a.c. effect will manifest itself through a peak in *S*_*I*_ at the frequency *ν*=*eV*_b_/*h* of amplitude ~*e*^2^/*τ*_F_ and quality factor *Q*≈*eV*_b_*τ*_F_/*h* (number of oscillations inside the transient regime). For example, a value of *τ*_F_=1 ns with an impedance of *Z*_c_=1 kΩ corresponds to a signal *S*_*I*_≈10^−29^ AHz^−1^, or a RF power *Z*_c_*S*_I_≈10^−26^ WHz^−1^, or equivalently to an effective noise temperature of *Z*_c_*S*_I_/*k*_B_≈1 mK, a signal well within reach with current cryogenic RF amplifiers (effective noise of 1–10 K). One can also try to observe the ac signal at larger, say optical, frequencies. This corresponds to lower impedances *Z*_c_≈377 Ω and a much lower number of photons *N*(*ν*)≈10^−8^ photons. Assuming a 10% efficiency (this will strongly depend on the geometry) and a 1 GHz repetition rate, we arrive at a few 1–10 photons per second. Note that all these estimates assume a single-channel geometry. For incoherent measurements, one can use multiple channels *N*_ch_~*S*/*λ*_F_^2^ (*S*: transverse surface of a Fabry–Perot cavity, *λ*_F_: Fermi wavelength) in parallel and increase the a.c. signal accordingly.

We now turn to a few concrete systems. We start with the Mach–Zehnder interferometer in the quantum Hall regime, as studied above, where the time of flight *τ*_F_ can be rather large. Indeed, its phase coherence length has been measured to be as high as 20 μm at low temperature (20 mK; ref. 10), which sets the upper limit for the difference of lengths between the two arms of the interferometer. Using typical values for the group velocity of the edge states of 10^4^–10^5^ ms^−1^, we arrive at *τ*_F_≈0.1–1 ns. One must therefore raise the potential at frequencies faster than 1–10 GHz to trigger the transient oscillations of amplitude ≈1 nA. Another interesting geometry is the ‘flying qubit’ experiment of ref. 24. This setup is essentially equivalent to a Mach–Zehnder interferometer but the two paths are not spatially resolved, see ref. 25. It could be advantageous with respect to the former Mach–Zehnder as the longitudinal velocities can be tuned using various gates so that *τ*_F_ can take much larger values. The Fabry–Perot cavity also offers a valid alternative either in its single-channel form (as implemented in a carbon nanotube or in the quantum Hall regime) or in a multichannel geometry. A small pillar made of epitaxial metals, say Cu-Ag-Cu, similar to the perpendicular structures used to fabricate spin valves would implement a many channels Fabry–Perot cavity with the metallic interfaces serving as the semi-transparent mirrors. A 1-μm^2^ pillar corresponds to *N*_ch_≈10^8^ and a corresponding increase in the signal. These are only possible geometries but many others could be considered as well, including systems, which are not ‘well defined’ interferometers. For instance the universal conductance fluctuations present in disordered systems also come from the interference of different trajectories of various lengths which should therefore lead to a transient a.c. current.

## Methods

### Numerical method

The time-dependent numerical calculations have been performed using the T-KWANT algorithm described in ref. 11, following the model detailed in ref. 12. The d.c. calculations were performed with the KWANT open source package^26^.

## Author contributions

X.W. initiated the project. B.G. and J.W. performed the numerical simulations. X.W., B.G. and J.W. performed the analytical calculations, data analysis and wrote the manuscript.

## Additional information

**How to cite this article:** Gaury, B. *et al*. Ac Josephson effect without superconductivity. *Nat. Commun*, 6:6524 doi: 10.1038/ncomms7524 (2015).
